# Risk of impaired school performance in children hospitalized with concussion: a population-based matched cohort study

**DOI:** 10.2217/cnc-2022-0012

**Published:** 2023-05-30

**Authors:** Reidar P Lystad, Anne McMaugh, Geoffrey Herkes, Gary Browne, Tim Badgery-Parker, Cate M Cameron, Rebecca J Mitchell

**Affiliations:** 1 Australian Institute of Health Innovation, Macquarie University, Macquarie Park NSW, 2109, Australia; 2 The Macquarie School of Education, Macquarie University, Macquarie Park NSW, 2109, Australia; 3 Sydney Medical School, University of Sydney, Camperdown NSW, 2006, Australia; 4 Royal North Shore Hospital, St Leonards NSW, 2065, Australia; 5 The Children's Hospital at Westmead, Westmead NSW, 2145, Australia; 6 Jamieson Trauma Institute, Royal Brisbane & Women's Hospital, Metro North Health, Herston QLD, 4029, Australia; 7 Centre for Healthcare Transformation, Australian Centre for Health Services Innovation, Queensland University of Technology, Brisbane QLD, 4000, Australia

**Keywords:** academic performance, concussion, high school completion, young people

## Abstract

**Aim:**

To examine the impact of concussion on objective measures of school performance.

**Materials & methods:**

Population-based matched cohort study using linked health and education records of young people aged ≤18 years hospitalized with concussion in New South Wales, Australia, during 2005–2018, and matched comparisons not hospitalized with any injury.

**Results:**

Young people with concussion had higher risk of not achieving the national minimum standards for literacy and numeracy assessments, ranging from 30% for numeracy to 43% for spelling, and not completing high school, ranging from 29% for year 10 to 77% for year 12, compared with matched peers.

**Conclusion:**

Young people hospitalized with concussion have impaired school performance compared with uninjured matched peers.

## Graphical abstract

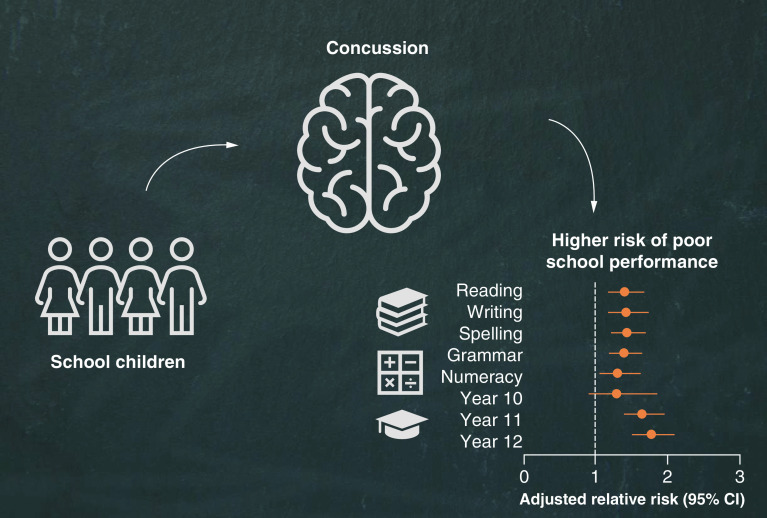

Concussion is a condition that exists along the clinical and pathological spectrum of traumatic brain injury (TBI) [1]. Concussion is a major public health concern because it is both common and associated with potential long-term sequelae [2,3]. About one in 80 people experience concussion every year, with higher incidence rates among contact sport athletes, military personnel, and children [4,5].

Concussion can manifest with a plethora of physical (e.g., headache, neck pain, dizziness, blurred vision, sensitivity to light or noise), cognitive (e.g., confusion, difficulty remembering, poor concentration, poor attention), emotional (e.g., irritability, depression, anxiety), and sleep-related (e.g., insomnia, drowsiness, fatigue) symptoms [1]. Although symptoms often resolve within weeks, about 35% of children with concussion experience symptoms that persist beyond four weeks [6]. Whether transient or persistent, concussion can have a negative impact on psychosocial and emotional health and quality of life of young people [7–9]. In addition to increased healthcare service use and school absenteeism [10], young people with concussion can also experience difficulties with functioning and learning at school [11,12]. Learning difficulties can lead to poor school performance, which can adversely affect long-term social outcomes and quality of life, including tertiary education attainment, career prospects, employment, income, and poverty in adulthood [13,14].

A systematic review concluded that although young people with concussion missed more school than those without head injury, there was minimal impact on school grades and national examination scores [15]. However, very few studies have examined the impact of concussion on objective measures of school performance (e.g., standardized tests and high school completion), which are generally considered to be more valid, reliable, and unbiased compared with subjective measures (i.e., student, teacher, and parental ratings of performance). Of the nine included studies, only five directly examined school performance outcomes, of which only two studies reported on standardized test results (i.e., national examinations) and only one study reported on high school completion [16–18]. Neither of the two studies examining standardized test results had matched comparisons, and both had relatively modest samples sizes of concussed children (i.e., n = 132 and n = 30) [16,17]. The one study reporting on high school completion did not find a statistically significant difference in odds of on-time graduation [18].

There is a need for large population-based cohort studies examining the impact of concussion on school performance, especially objective measures of school performance (e.g., standardized national school assessments and high school completion), while accounting for potential moderating factors (e.g., sex, age, socioeconomic status, and comorbid health conditions). Thus, this study aimed to compare standardized test performance and high school completion in a large population-based sample of young people hospitalized with concussion and matched peers not hospitalized with any injury.

## Materials & methods

### Study design

This was a population-based matched case-comparison cohort study using linked health, education, birth, and mortality data of young people aged ≤18 years in New South Wales (NSW), Australia, during 1 January 2005 to 31 December 2018. Ethical approval and a waiver of consent was obtained from the NSW Population and Health Services Research Ethics Committee (reference number 2018HRE0904), and a research protocol has been published previously [19].

### Data sources

Information on health service use was obtained from NSW emergency department (ED) and hospital admission data collections. ED visits to public hospitals included data on arrival and departure times, visit type, and provisional diagnosis. Hospital admissions were to public or private hospitals, and contained information on demographics, diagnoses, separation type (e.g., hospital transfer, discharge, death), and clinical procedures. Mortality data was obtained from the NSW Registry of Births, Deaths and Marriages and young people who died during the study period were excluded from the analyses.

School performance and parental demographic information were obtained from National Assessment Plan for Literacy and Numeracy (NAPLAN) assessments conducted annually in May from 2008 to 2018 for government, Catholic, and independent schools. NAPLAN assessments were conducted in school years 3 (i.e., 7–9 years of age), 5 (i.e., 9–11 years of age), 7 (i.e., 11–13 years of age), and 9 (i.e., 13–15 years of age). NAPLAN tests included assessments in reading, writing, spelling, grammar, and numeracy, and each NAPLAN test score was converted into a 10-band scale that indicate whether performance was above, at, or below the national minimum standard (NMS) [20]. A young person's attendance, absence, withdrawal (e.g., philosophical objections to testing or religious beliefs) or exemption due to significant disability for the NAPLAN assessments was obtained (Supplementary Table 1). There were no significant differences in proportions of cases and comparisons who were absent or withdrawn from NAPLAN assessments in any of the school years. Young people exempt from sitting an assessment due to severe disability or language difficulties were rated as scoring below the NMS as per technical guidelines [20]. From 2011, the writing assessment task changed from a narrative to a persuasive writing task. Because the results from narrative and persuasive writing tasks were not comparable, pre-2011 narrative writing task results were excluded from the analyses. A young person was classified as having a language background other than English (LBOTE) if either they or their parents or guardians spoke a language other than English at home [20]. Where there were multiple records of the parents' level of education (e.g., ≤year 12; certificate I-IV, trade or diploma; bachelor or higher degree; or not stated or unknown) and occupation (e.g., senior manager/qualified professional; business manager/associate professional; trades/clerks/skilled office/sales or service; machine operators/hospitality/assistants/laborers; or not in paid work in last 12 months), the highest level of education and occupation of either parent was identified.

Information on high school completion for year 10 (i.e., 15–16 years of age), year 11 (i.e., 16–17 years of age), and year 12 (i.e., 17–18 years of age) from 2005 to 2018 were obtained from the Record of School Achievement and the Higher School Certificate issued by the NSW Education Standards Authority. High school year completions were recorded in medio/ultimo December each year.

### Cohort selection criteria

The case cohort comprised young people with a year of birth ≥1997 who were aged ≤18 years at index hospitalization with a principal diagnosis of concussion (International Classification of Diseases, 10th Revision, Australian Modification [ICD-10-AM]: S06.0) during 1 January 2005 to 31 December 2018. Cases were included in school years 3, 5, 7, and 9 cohorts if the young person completed all NAPLAN assessments in a school year and was hospitalized with concussion prior to the NAPLAN assessment period in a school year. Cases were included in high school years 10–12 cohorts if the young person was hospitalized with concussion prior to the recording of high school completion in a high school year.

The comparison cohort comprised young people who were not hospitalized with any injury during 1 July 2001 to 31 December 2018. The comparisons were randomly selected from NSW birth records and matched 1:1 on age, sex, and residential postcode. The selection timeframe for comparisons included a 3.5-year wash-out period prior to the case selection timeframe to avoid the potential selection of comparisons who may have been hospitalized with any injury prior to the case criteria timeframe.

The Centre for Health Record Linkage identified the comparison cohort and linked the birth, health, education and mortality records using probabilistic record linkage. Upper and lower probability cut-offs for a link were 0.75 and 0.25 and record groups with probabilities between the cut-offs were clerically reviewed.

### Geographical location & socioeconomic status

The Australian Statistical Geographical Standard [21], which is based on distance to service centers, was used to classify the residential postcode of the young person as either urban (i.e., major cities) or rural (i.e., inner and outer regional, remote, and very remote). Residential postcode was also mapped to the Index of Relative Socioeconomic Disadvantage and partitioned into quintiles from most (i.e., 1) to least (i.e., 5) disadvantaged [22]. The remoteness area of the school was obtained from NAPLAN records and was categorized as major city, inner regional, outer regional, or remote [21].

### Identification of chronic health conditions

Common chronic health conditions for young people were identified from prior studies of pediatric comorbidities [23,24]. For this study, chronic health conditions were identified using a 3-year look-back period from the index hospitalization date and ICD-10-AM diagnosis codes in up to 50 additional diagnosis fields (Supplementary Table 2).

### Data management & analysis

Data analysis was conducted using SAS 9.4 (SAS Institute, Cary NC). All hospital episodes of care related to the same event were linked to form a period of care. Chi-square tests were used to compare characteristics of cases and matched peers. The number of ED visits, hospital admissions, and hospital length of stay (LOS) (measured in days) during and after the index admission were identified for cases and matched peers and compared using Mann-Whitney U tests. The calculation of hospital LOS was cumulative and included transfers between hospitals. All chi-square and Mann-Whitney-U tests were two-tailed with an alpha level set at 0.05.

Generalized linear mixed modelling was used to examine risk of not achieving the NMS for each NAPLAN assessment for cases and matched peers across school years 3, 5, 7 and 9. Each multilevel model was fitted using a binary distribution, log link function, and Kenward-Roger approximation of denominator degrees of freedom. The residual option of the random statement was used to model R-side covariance and data were analyzed to account for within student correlation in the longitudinal data and repeated measurements using an autoregressive covariance structure. Generalized linear modelling was used to examine risk of not completing high school years 10–12 for cases and matched peers. Each generalized linear model was fitted using generalized estimating equations with binomial distribution and a log link function. All generalized linear mixed models and generalized linear models were fitted using mixed selection method (i.e., combined forward selection and backward elimination), whereby non-collinear covariates were sequentially added to and removed from the model, with p-value thresholds set at 0.05 to enter and 0.10 to leave the model. The variables included in each final fitted model are listed in the footnotes of Supplementary Table 3. Cases and matched peers with missing values for socioeconomic status and language background were excluded from the model fitting procedures. As young people in the comparison cohort could have nil hospital LOS, a small constant value was added to hospital LOS before log transformation. Adjusted relative risks (ARR) with 95% confidence intervals (CI) were derived from each final fitted model. The family-wise error rate arising from multiple comparisons (i.e. five NAPLAN assessments) was controlled for using the Bonferroni-Holm procedure.

## Results

The number of young people hospitalized with concussion where a matched comparison could be identified was n = 1049 for year 3; n = 1035 for year 5; n = 932 for year 7; n = 689 for year 9; n = 1445 for year 10; n = 1366 for year 11; and n = 1182 for year 12. For each school year, there were more young males than females (range 58.2% to 69.2% vs 30.8% to 41.8%, respectively). More than half of young people with concussion were from urban areas (range 55.9% to 60.3%). Compared with matched peers, young people with concussion were significantly less likely to have chronic health conditions, although chronic health conditions were very uncommon among both groups (Tables 1 & 2). Healthcare service use was significantly higher for young people hospitalized with concussion then matched peers across all school years (Table 3).

**Table 1.** Demographic, parental, and school characteristics of young people hospitalized with concussion and matched peers not hospitalized with any injury by school year, linked health and school performance data from New South Wales, 2005–2018.

Characteristics	Year 3^†^				Year 5^‡^				Year 7^§^				Year 9^¶^			
	Case (n = 1049)		Comparison (n = 1049)		Case (n = 1035)		Comparison (n = 1035)		Case (n = 932)		Comparison (n = 932)		Case (n = 689)		Comparison (n = 689)	
	n	%	n	%	n	%	n	%	n	%	n	%	n	%	n	%
Sex																
– Male	610	58.2	610	58.2	641	61.9	641	61.9	613	65.8	613	65.8	477	69.2	477	69.2
– Female	439	41.8	439	41.8	394	38.1	394	38.1	319	34.2	319	34.2	212	30.8	212	30.8
Location of residence^#^																
– Urban	606	57.8	606	57.8	607	58.7	607	58.7	549	58.9	549	58.9	395	57.3	395	57.3
– Rural	442	42.1	442	42.1	427	41.3	427	41.3	382	41.0	382	41.0	292	42.4	292	42.4
Socioeconomic status^#^																
– 1 (most disadvantaged)	221	21.1	221	21.1	229	22.1	229	22.1	200	21.5	200	21.5	134	19.5	134	19.5
– 2	273	26.0	273	26.0	255	24.6	255	24.6	213	22.9	213	22.9	172	25.0	172	25.0
– 3	247	23.6	247	23.6	239	23.1	239	23.1	221	23.7	221	23.7	153	22.2	153	22.2
– 4	99	9.4	99	9.4	100	9.7	100	9.7	97	10.4	97	10.4	82	11.9	82	11.9
– 5 (least disadvantaged)	208	19.8	208	19.8	211	20.4	211	20.4	200	21.5	200	21.5	146	21.2	146	21.2
LBOTE																
– Non-LBOTE	872	83.1	853	81.3	871	84.2	856	82.7	806	86.5	779	83.6	596	86.5	580	84.2
– LBOTE	171	16.3	194	18.5	160	15.5	178	17.2	126	13.5	152	16.3	92	13.4	109	15.8
– Not known	6	0.6	2	0.2	4	0.4	1	0.1	0	0.0	1	0.1	1	0.2	0	0.0
Health conditions																
– 0	1046	99.7	1037	98.9	1032	99.7	1022	98.7	930	99.8	922	98.9	686	99.6	678	98.4
– ≥1	3	0.3	12	1.1	3	0.3	13	1.3	2	0.2	10	1.1	3	0.4	11	1.6
Parent highest level of education																
– Year 11 or equivalent	67	6.4	78	7.4	88	8.5	101	9.8	64	6.9	79	8.5	47	6.8	53	7.7
– Year 12 or equivalent	49	4.7	28	2.7	61	5.9	38	3.7	44	4.7	31	3.3	37	5.4	31	4.5
– Certificate I-IV or trade	316	30.1	351	33.5	341	33.0	348	33.6	307	32.9	311	33.4	224	32.5	223	32.4
– Advanced diploma/ diploma	163	15.5	160	15.3	157	15.2	165	15.9	159	17.1	163	17.5	124	18.0	127	18.4
– Bachelor degree or higher	399	38.0	379	36.1	374	36.1	364	35.2	351	37.7	332	35.6	252	36.6	236	34.3
– Not stated/not known	55	5.2	53	5.1	14	1.4	19	1.8	7	0.8	16	1.7	5	0.7	19	8.8
Parent highest occupation																
– Senior manager/qualified professional	333	31.7	310	29.6	323	31.2	304	29.4	307	32.9	306	32.8	230	33.4	224	32.5
– Business management/ associate professional	246	23.5	259	24.7	260	25.1	254	24.5	259	27.8	232	24.9	196	28.5	184	26.7
– Trades, clerks, skilled office, sales and service	225	21.5	233	22.2	222	21.5	223	21.6	194	20.8	210	22.5	148	21.5	145	21.0
– Machine operators, hospitality, assistants, laborers	153	15.6	144	13.7	150	14.5	151	14.6	121	13.0	118	12.7	69	10.0	86	12.5
– Not in paid work in last 12 months	65	6.2	81	7.7	57	5.5	81	7.8	41	4.4	53	5.7	33	4.8	31	4.5
– Not known	27	2.6	22	2.1	23	2.2	22	2.1	10	1.1	13	1.4	13	1.9	19	2.8
School sector																
– Government	735	70.2	745	71.0	712	68.9	709	68.5	511	54.8	550	59.0	372	54.0	397	57.6
– Catholic	217	20.7	215	20.5	214	20.7	230	22.2	266	28.5	235	25.2	216	31.4	182	26.4
– Independent	95	9.1	89	8.5	108	10.4	96	9.3	155	16.6	147	15.8	101	14.7	110	16.0
Remoteness area of school^††^																
Major city	584	55.8	618	58.9	587	56.8	621	60.0	560	60.1	567	60.8	409	59.4	402	58.4
Inner regional	326	31.1	308	29.4	307	29.7	284	27.4	276	29.6	274	29.4	215	31.2	225	32.7
Outer regional/remote	137	13.1	123	11.7	140	13.5	130	12.6	96	10.3	91	9.8	65	9.4	62	9.0

† Year 3 chi-square tests: LBOTE p = 0.161; Health conditions p = 0.017; Parent highest level of education p = 0.110; Parent highest occupation p = 0.574; School sector (excluding 2 home-schooled) p = 0.874; Remoteness area of school (excluding 2 missing) p = 0.329.

‡ Year 5 chi-square tests: LBOTE p = 0.236; Health conditions p = 0.011; Parent highest level of education p = 0.193; Parent highest occupation p = 0.435; School sector (excluding 1 home-schooled) p = 0.525; Remoteness area of school (excluding 1 missing) p = 0.329.

§ Year 7 chi-square tests: LBOTE p = 0.143; Health conditions p = 0.021; Parent highest level of education p = 0.159; Parent highest occupation p = 0.538; School sector p = 0.168; Remoteness area of school p = 0.912.

¶ Year 9 chi-square tests: LBOTE p = 0.265; Health conditions p = 0.032; Parent highest level of education p = 0.087; Parent highest occupation p = 0.617; School sector p = 0.129; Remoteness area of school p = 0.836.

# Location of residence and socioeconomic status were missing for 1 case and comparison in year 3; 1 case and comparison in year 5; 1 case and comparison in year 7; and 2 cases and comparisons in year 9.

†† Remoteness area of school was undefined for home schooled children: 2 cases in year 3 and 1 case in year 5.

LBOTE: Language background other than English; NSW: New South Wales.

**Table 2.** Demographic, parental, and school characteristics of young people hospitalized with concussion and matched peers not hospitalized with any injury by high school year, linked health and school performance data from New South Wales, 2005–2018.

Characteristics	Year 10^†^				Year 11^‡^				Year 12^§^			
	Case (n = 1445)		Comparison (n = 1445)		Case (n = 1366)		Comparison (n = 1366)		Case (n = 1182)		Comparison (n = 1182)	
	n	%	n	%	n	%	n	%	n	%	n	%
Sex:												
– Male	996	68.9	996	68.9	938	68.7	938	68.7	800	67.7	800	67.7
– Female	449	31.1	449	31.1	428	31.3	428	31.3	382	32.3	382	32.3
Location of residence^¶^												
– Urban	807	55.9	807	55.9	809	59.2	809	59.2	713	60.3	713	60.3
– Rural	636	44.0	636	44.0	555	40.6	555	40.6	468	39.6	468	39.6
Socioeconomic status^¶^												
– 1 (most disadvantaged)	283	19.6	283	19.6	263	19.3	263	19.3	230	19.5	230	19.5
– 2	392	27.1	392	27.1	353	25.8	353	25.8	290	24.5	290	24.5
– 3	349	24.2	349	24.2	337	24.7	337	24.7	294	24.9	294	24.9
– 4	141	9.8	141	9.8	148	10.8	148	10.8	123	10.4	123	10.4
– 5 (least disadvantaged)	278	19.2	278	19.2	263	19.3	263	19.3	244	20.6	244	20.6
LBOTE												
– Non-LBOTE	753	52.1	713	49.3	643	47.1	597	43.7	466	39.2	427	36.1
– LBOTE	115	8.0	111	7.7	99	7.3	91	6.7	84	7.1	71	6.0
– Not known	577	39.9	621	43.0	624	45.7	678	49.6	632	53.5	684	57.9
Health conditions												
– 0	1433	99.2	1412	97.7	1353	99.1	1333	97.6	1170	99.0	1151	97.4
– ≥1	12	0.8	33	2.3	13	1.0	33	2.4	12	1.0	31	2.6
Parent highest level of education												
– Year 11 or equivalent	81	5.6	77	5.3	62	4.5	65	4.8	54	4.6	38	3.2
– Year 12 or equivalent	43	3.0	43	3.0	37	2.7	39	2.9	33	2.8	31	2.6
– Certificate I–IV or trade	283	19.6	263	18.2	236	17.3	209	15.3	150	12.7	139	11.8
– Advanced diploma/ diploma	153	10.6	151	10.5	138	10.1	124	9.1	111	9.4	94	8.0
– Bachelor degree or higher	298	20.6	267	18.5	262	19.2	230	16.8	194	16.4	179	15.1
– Not stated/not known	644	40.6	644	44.6	631	46.2	699	51.2	640	54.2	701	59.3
Parent highest occupation												
– Senior manager/qualified professional	271	18.8	246	17.0	226	16.5	209	15.2	167	14.3	166	14.0
– Business management/ associate professional	232	16.1	236	16.3	213	15.6	203	14.9	160	13.5	148	12.5
– Trades, clerks, skilled office, sales and service	176	12.2	167	11.6	155	11.4	141	10.3	113	9.6	97	8.2
– Machine operators, hospitality, assistants, laborers	116	8.0	113	7.8	90	6.6	85	6.2	64	5.4	52	4.4
– Not in paid work in last 12 months	47	3.3	41	2.8	40	2.9	29	2.1	31	2.6	17	1.4
– Not known	603	41.7	642	44.4	642	47.0	699	51.2	647	54.7	702	59.4
School sect**or**												
– Government	496	34.3	499	34.5	418	30.6	399	29.2	300	25.4	279	23.6
– Catholic	203	14.5	245	17.0	216	15.8	175	12.8	167	14.1	134	11.3
– Independent	121	8.4	116	8.0	102	7.5	110	8.1	78	6.6	82	6.9
– Not known	625	43.3	585	40.5	630	46.1	682	49.9	637	53.9	687	58.1

† Year 10 chi-square tests: LBOTE p = 0.249; Health conditions p = 0.002; Parent highest level of education p = 0.394; Parent highest occupation p = 0.677; School sector p = 0.146.

‡ Year 11 chi-square tests: LBOTE p = 0.118; Health conditions p = 0.003; Parent highest level of education p = 0.153; Parent highest occupation p = 0.317; School sector p = 0.069.

§ Year 12 chi-square tests: LBOTE p = 0.089; Health conditions p = 0.004; Parent highest level of education p = 0.153; Parent highest occupation p = 0.099; School sector p = 0.095.

¶ Location of residence and socioeconomic status were missing for 2 cases and comparisons in year 10; 2 cases and comparisons in year 11; and 1 case and comparison in year 12.

LBOTE: Language background other than English; NSW: New South Wales.

**Table 3.** Healthcare service use for young people hospitalized with concussion and matched peers not hospitalized with any injury by school year, linked health and school performance data from New South Wales, 2005–2018.

	Case		Comparison		p-value^†^
	Mean	SD	Mean	SD	
Number of emergency department visits:					
Year 3 (n = 1049 matched pairs)	7.7	7.3	3.9	4.6	<0.001
Year 5 (n = 1035 matched pairs)	7.8	7.3	4.0	4.6	<0.001
Year 7 (n = 932 matched pairs)	7.7	6.8	3.7	4.3	<0.001
Year 9 (n = 689 matched pairs)	7.1	6.2	3.6	4.3	<0.001
Year 10 (n = 1445 matched pairs)	7.5	7.1	4.5	4.3	<0.001
Year 11 (n = 1366 matched pairs)	6.9	6.8	4.1	4.0	<0.001
Year 12 (n = 1182 matched pairs)	6.4	6.4	4.0	4.1	<0.001
Number of hospital admissions:					
Year 3 (n = 1049 matched pairs)	2.8	2.5	1.5	12.1	<0.001
Year 5 (n = 1035 matched pairs)	2.8	2.8	1.4	12.3	<0.001
Year 7 (n = 932 matched pairs)	2.7	2.4	1.0	1.9	<0.001
Year 9 (n = 689 matched pairs)	2.5	2.3	0.8	1.9	<0.001
Year 10 (n = 1445 matched pairs)	2.6	2.7	2.2	7.7	<0.001
Year 11 (n = 1366 matched pairs)	2.5	2.4	2.2	8.6	<0.001
Year 12 (n = 1182 matched pairs)	2.4	2.4	1.8	2.0	<0.001
Cumulative hospital length of stay (days):					
Year 3 (n = 1049 matched pairs)	5.5	10.3	3.6	13.6	<0.001
Year 5 (n = 1035 matched pairs)	5.7	11.0	3.6	14.2	<0.001
Year 7 (n = 932 matched pairs)	4.8	7.2	2.7	6.0	<0.001
Year 9 (n = 689 matched pairs)	4.3	7.1	1.9	4.3	<0.001
Year 10 (n = 1445 matched pairs)	5.5	21.9	1.8	6.6	<0.001
Year 11 (n = 1366 matched pairs)	3.7	13.9	1.4	6.5	<0.001
Year 12 (n = 1182 matched pairs)	3.3	14.3	1.1	3.3	<0.001

† Mann-Whitney U test.

NSW: New South Wales; SD Standard deviation.

**Figure 1.**
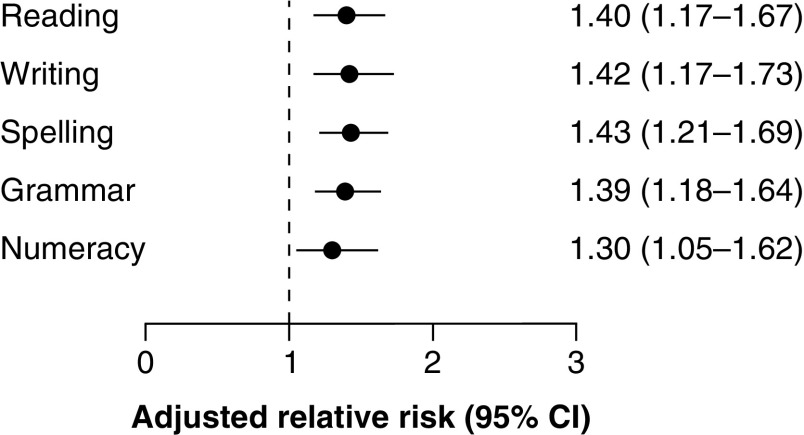
Adjusted relative risk of not achieving the national minimum standard for each National Assessment Plan for Literacy and Numeracy assessment in school years 3, 5, 7, and 9 for young people hospitalized with concussion compared with matched peers not hospitalized with any injury. Adjusted relative risks with 95% CIs of not achieving the national minimum standard for each NAPLAN assessment in school years 3, 5, 7, and 9 were derived from generalized linear mixed models using linked health and school performance data from New South Wales, 2005–2018.

For school years 3 to 9, the proportion of young people with concussion who did not achieve the NMS for NAPLAN assessments ranged from 6.8% to 9.1% for reading, 6.2% to 20.5% for writing, 7.5% to 13.2% for spelling, 7.7% to 12.6% for grammar, and 3.3% to 6.8% for numeracy (Supplementary Table 4). Multilevel modelling identified that young people with concussion had higher risk of not achieving the NMS for all five NAPLAN assessments compared with matched peers, with elevated risks ranging from 30% for numeracy (ARR 1.30; 95%CI 1.05–1.62) to 43% for spelling (ARR 1.43; 95%CI 1.21–1.69) (Figure 1 & Supplementary Table 3).

**Figure 2.**
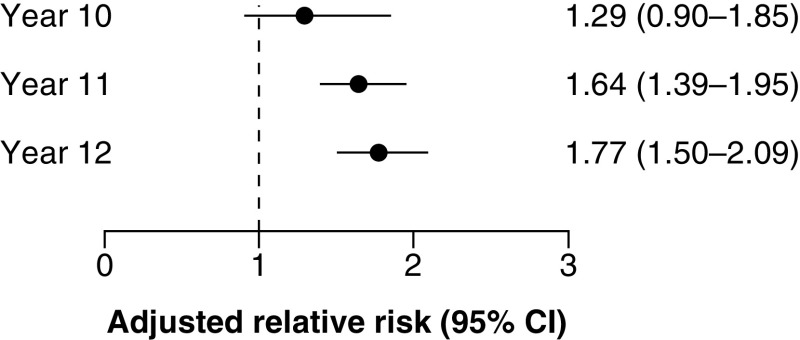
Adjusted relative risks of not completing high school years 10, 11 and 12 for young people hospitalized with concussion compared with matched peers not hospitalized with any injury. Adjusted relative risks with 95% confidence intervals derived from generalized linear models of linked health and school performance data from New South Wales, 2005–2018.

The proportion of young people with concussion who did not complete high school was 4.6% for year 10, 25.5% for year 11, and 32.9% for year 12 (Supplementary Table 4). Young people with concussion had 29% higher risk of not completing year 10 (ARR 1.29; 95%CI 0.90–1.85), 64% higher risk of not completing year 11 (ARR 1.64; 95%CI 1.39–1.95), and 77% higher risk of not completing year 12 (ARR 1.77; 95%CI 1.50–2.09) compared with matched peers (Figure 2 & Supplementary Table 3).

## Discussion

This large population-based matched cohort study found that young people hospitalized with concussion had a higher risk of not achieving minimum standards in national standardized assessments of literacy and numeracy, as well as not completing high school, compared with matched peers. These findings contradict a previous review that concluded the effect of concussion or mild TBI on academic performance was negligible and non-significant at the group-level [15]. The conclusion of this previous review was based on few studies directly examining measures of school performance.

Firstly, only three studies have previously examined the effect of concussion or mild TBI on teacher-assigned school grades. Of these, one study reported no significant effect on school grades [25], while two studies found statistically significant decreases in some school subjects but not in others [18,26]. More recently, another study reported no significant effect on high school grade point averages [27]. The current study did not examine teacher-assigned grades because they are known to be prone to a wide range of biases [28]. For instance, teacher bias in grading has been linked to student gender, race and ethnicity, socioeconomic status, perceived attractiveness, and prior academic performance [29]. It is possible that teachers either unconsciously or consciously lower their expectations for students with concussion.

Secondly, only two studies examined the effect of mild TBI on standardized test scores. While both studies reported poorer examination results among young people with mild TBI, neither detected statistically significant group differences [16,17]. Both studies featured relatively small cohorts of young people with mild TBI (i.e., n = 30 and n = 132), and the resulting lack of statistical power may have precluded them from detecting statistically significant small or moderate effect sizes (i.e., relative risks of 1.22–1.85 and 1.86–3.00, respectively) [29]. In contrast, the current study featured a relatively large cohort of young people with concussion (i.e., n = 3689 across years 3–9), which allowed detection of statistically significant small effect sizes. Differences in standardized school performance measures across studies may also have contributed to the divergent findings.

Thirdly, only one study examined the effect of concussion on high school completion. It found the proportion of young people with concussion who did not graduate from high school on time was slightly higher than young people without injury (i.e., 25.3 vs 23.0%, respectively), but the difference was not statistically significant (adjusted odds ratio 1.19 [95%CI 0.98–1.36]) [18]. The current study detected significant small effect sizes for not completing high school years 11 and 12 (i.e., 1.64 [95% CI: 1.39–1.95] and 1.77 [95% CI: 1.50–2.09], respectively), but not year 10 (i.e., 1.29 [95%CI 0.90–1.85]). The reason for the differences between the previous and present studies is unclear, but may be related to differences in study designs, study settings, and cohort selections. That is, Russell and colleagues [18] examined on-time graduation in a cohort of young people in Manitoba, Canada, from 2005–2006 to 2010–2011, whereas the current study examined completion of each high school year in a cohort of young people in NSW, Australia, from 2005 to 2018. There may be differences between Canadian and Australian high school settings that could result in differential mitigation of concussion symptoms and sequelae (e.g., school-based accommodations and individualized care plans). In addition, high school completion rates in the general student population have increased over time [30,31], which may have improved the ability of analyses to detect the potential effect of concussion on high school completion. It is also important to note that a larger proportion of young people leave high school after the last mandatory year of schooling (i.e., year 10) to pursue trades or apprenticeships, which may explain why the observed effects in the current study were smaller for year 10 than years 11 and 12.

The underlying mechanisms leading to impaired school performance among young people with concussion were not examined in this study, but is likely attributable to several factors. Firstly, concussion typically results in school absenteeism, and thus lost opportunities for learning [15]. Although data on school absences were not available, the current study found significantly higher hospital LOS among young people with concussion compared with matched peers not hospitalized with any injury. This is consistent with previous studies demonstrating significantly higher school absenteeism among young people with concussion [10,32]. Secondly, concussion symptoms include a wide range of neurocognitive deficits, including poorer attention and concentration [33,34], working and short-term memory [33,35], cognitive processing speed and reaction time [33–35], and verbal fluency [33]. These neurocognitive deficits can become a significant barrier to effective learning and, consequently, academic performance. Thirdly, sleep disturbances are common after concussion [36], and studies have demonstrated that sleep problems in young people are associated with impaired academic performance [37,38].

The findings herein suggest there is a need for further research to mitigate adverse educational outcomes in young people with concussion, including the identification and implementation of effective strategies. Young people with concussion, especially those with, or at risk of developing, persistent symptoms may benefit from multidisciplinary management and better care coordination between acute, primary, tertiary, and specialist healthcare settings, as well as school-based accommodations and early interventions to prevent adverse outcomes, such as early school leaving [39,40]. It is vital that the school sector recognizes the gravity of the concussion problem and adopts a strong commitment to health and safety. That includes improving policies, procedures, and protocols for returning to learning following concussion and implementing strategies to prevent concussion in high-risk activities, such as school- and community-based contact and collision sports. Although return-to-learn protocols, accommodations, and interventions have been developed for young people with concussion [12,41], it is not always clear how these should be operationalized and effectively implemented in school settings. Previous research has found considerable variability in compliance with return-to-learn recommendations in school settings [42]. Schools have an obligation to support al^44^l students with learning support needs, and they typically adopt a multi-tiered support framework with ascending levels of academic support for students who require it [43]. Effective implementation of return-to-learn protocols in schools may depend on how well they are integrated into existing multi-tiered support frameworks.

### Strengths & limitations

The strengths of this study were that it was a large population-based study linking health and educational records over a 14-year period, and that it was able to control for several potential confounding variables. However, there were some limitations. Cases were identified from hospital admission records, and thus did not include young people with concussion who presented solely to primary care or allied healthcare professionals. The number of identified cases in each school year depended on several factors, including the size of the population at risk (which changes over time due general population growth and attrition) and differences in concussion incidence rates across age groups (i.e., higher rates typically observed among older children) [44,45]. There was no information on the external cause and activity at the time of injury, severity of concussion symptoms, concussion management, potential moderating factors (e.g., school absences and school accommodations), or the actual date a young person might have dropped out of school, and future studies are recommended to examine these factors. Although it was possible for cases to have experienced more than one concussion during the study period, stratified analyses and model adjustments for multiple concussions were precluded by the small number of cases who had more than one concussion during the study period (i.e., n = 65 across all school years). Because only comorbidities relevant to a hospitalization are indicated in diagnosis classifications, it is possible that some comorbid conditions were not identified. However, using a relatively long look-back period (i.e., 3 years) reduced the likelihood of under-ascertainment of relevant chronic health conditions in the hospitalization records. Residential postcode was missing for a small number of cases (see Tables 1 & 2 footnotes for more details), which precluded assigning socioeconomic status for these cases. Visits to private hospital EDs were not able to be accessed for this study; however, these represent only a small proportion of the total number of injury-related ED visits in Australia.

## Conclusion

Young people hospitalized with concussion have higher risk of not achieving minimum standards for literacy and numeracy, as well as not completing high school, compared with matched peers not hospitalized with any injury. Improved multidisciplinary management, care coordination, and appropriately implemented return-to-learn protocols in schools are vital to minimize the adverse effects of concussion on school performance and its potential sequelae, such as early school leaving, unemployment and poverty in adulthood.

**Summary points**

- Although concussion is associated with school absenteeism and difficulties with functioning and learning at school, there is scarce evidence regarding its potential impact on objective measures of school performance.
- This large population-based matched cohort study used linked health and educational records to compare school performance of young people aged ≤18 years hospitalized with concussion and matched peers not hospitalized with any injury (N = 7,689 matched pairs).
- Young people hospitalized with concussion have significantly higher risk of not achieving national minimum standards for literacy and numeracy in school years 3–9, compared with matched peers not hospitalized with any injury.
- Young people hospitalized with concussion have significantly higher risk of not completing high school years 11 and 12, compared with matched peers not hospitalized with any injury.
- There is a need to improve learning support systems and concussion recovery management to mitigate impaired school performance in young people with concussion.
