# Supplementary material for: Risk of impaired school performance in children hospitalized with concussion: a population-based matched cohort study
Source: Concussion. 2023 May 30;8(3):CNC105. doi: 10.2217/cnc-2022-0012 (PMC10488614; doi:10.2217/cnc-2022-0012)
Supplement: Supplementary file 1 [file cnc-08-105-s1.pdf]

Supplemental Material

Supplementary Table 1. Frequency and proportion of young people hospitalized with concussion and matched peers not hospitalized with any injury absent or withdrawn from a NAPLAN assessment by school year, linked health and school performance data NSW, 2005–2018.

Supplementary Table 2. Health conditions and ICD-10-AM classifications.

Supplementary Table 3. Unadjusted and adjusted relative risks of not achieving NMS on NAPLAN assessments and not completing high school for young people hospitalized with concussion compared to matched peers not hospitalized with any injury by school year, linked health and school performance data NSW, 2005–2018.

Supplementary Table 4. School performance outcomes for young people hospitalized with concussion and matched peers not hospitalized with any injury by school year, linked health and school performance data NSW, 2005–2018.

Supplementary Table 1. Frequency and proportion of young people hospitalized with concussion and matched peers not hospitalized with any injury absent or withdrawn from a NAPLAN assessment by school year, linked health and school performance data NSW, 2005–2018.

	**Year 3**				**Year 5**				**Year 7**				**Year 9**			
	**Case**		**Comparison**		**Case**		**Comparison**		**Case**		**Comparison**		**Case**		**Comparison**	
	**No.**	**%**	**No.**	**%**	**No.**	**%**	**No.**	**%**	**No.**	**%**	**No.**	**%**	**No.**	**%**	**No.**	**%**
**Absent** [a]																
NAPLAN reading	65	1.9	48	1.6	56	1.9	42	1.6	75	3.1	64	3.1	120	6.5	77	5.1
NAPLAN writing [b]	46	2.1	35	1.8	47	1.9	39	1.7	67	2.8	61	2.9	104	5.7	68	4.5
NAPLAN spelling	58	1.7	46	1.5	55	1.9	37	1.4	60	2.5	60	2.9	105	5.7	69	4.6
NAPLAN grammar	58	1.7	46	1.5	55	1.9	37	1.4	60	2.5	60	2.9	105	5.7	69	4.6
NAPLAN numeracy	62	2.1	62	2.1	57	2.0	51	2.0	105	4.3	87	4.2	136	7.4	89	5.9
**Withdrawn** [a]																
NAPLAN reading	52	1.6	39	1.3	27	0.9	17	0.7	19	0.8	15	0.7	19	1.0	24	1.6
NAPLAN writing [b]	41	1.9	32	1.6	27	1.1	18	0.8	18	0.7	13	0.6	21	1.1	24	1.6
NAPLAN spelling	47	1.4	39	1.3	26	0.9	18	0.7	18	0.7	13	0.6	18	1.0	24	1.6
NAPLAN grammar	47	1.4	39	1.3	26	0.9	18	0.7	18	0.7	13	0.6	18	1.0	24	1.6
NAPLAN numeracy	46	1.4	39	1.3	26	0.9	17	0.7	19	0.8	15	0.7	21	1.1	24	1.6
**Exempted** [c]																
NAPLAN reading	19	1.8	12	1.1	10	1.0	9	0.9	11	1.2	6	0.6	9	1.3	5	0.7
NAPLAN writing [b]	19	1.8	12	1.1	10	1.0	9	0.9	11	1.2	6	0.6	9	1.3	5	0.7
NAPLAN spelling	18	1.7	12	1.1	10	1.0	9	0.9	11	1.2	6	0.6	9	1.3	5	0.7
NAPLAN grammar	18	1.7	12	1.1	10	1.0	9	0.9	11	1.2	6	0.6	9	1.3	5	0.7
NAPLAN numeracy	17	1.6	12	1.1	10	1.0	9	0.9	11	1.2	5	0.5	9	1.3	4	0.6

NAPLAN: National Assessment Plan for Literacy and Numeracy; NSW: New South Wales.

[a] Excluded from analyses. Proportions calculated using the number of cases and comparisons prior to excluding those who did not complete all five NAPLAN assessments as the denominator. There were no significant differences in proportions of cases and comparisons who were absent or withdrawn from NAPLAN assessments in any of the school years.

[b] NAPLAN writing assessment data pre-2011 were excluded.

[c] Rated as scoring below the national minimum standard, as per NAPALAN technical guidelines, and included in analyses. Proportions calculated using the number of cases and comparisons included in the analyses as the denominator.

Supplementary Table 2. Health conditions and ICD-10-AM classifications.

**Health condition**	**ICD-10-AM classification(s)**
**Circulatory system**	
Hypertension	I10-I15
**Digestive system and allergies**	
Celiac disease and other serious allergies	K52.2, K90.0, T78.0, T78.2, T78.4
**Endocrine, nutritional and metabolic conditions**	
Diabetes	E09-E14
Obesity	E66
Cystic fibrosis	E84
**Immune system conditions and coagulation defects**	
Anemia	D50-D53 and D55-D64
Coagulation defects (e.g., hemophilia)	D65-C68
**Mental health conditions**	
Autism spectrum disorders	F84
Behavioral and emotional disorders of childhood	F90-F98
Cognitive and behavioral delay	F80-F83 and F88-F89
Eating disorders	F50
Hyperkinetic disorder	F90
Mental retardation	F70-F79
Mood affective disorders	F30-F39
Neurotic, stress-related and somatoform disorders	F40-F48
Personality disorders	F60-F69
Schizophrenia, schizotypal and delusions disorders	F20-F29
**Neoplasms**	
All malignancies	C00-D48
Acute lymphoblastic leukemia and acute myeloid leukemia	C91.0, C92.0
Brain cancer	C71
**Nervous system conditions**	
Cerebral palsy	G80
Epilepsy	G40
**Renal conditions**	I12.0, I13.1, NO3, NO5, N18-N19, N25.0, Z49, Z94.0, Z99.2
**Respiratory conditions**	
Chronic lower respiratory disease	J40-J47
Asthma	J45

ICD-10-AM: International Statistical Classification of Diseases and Related Health Problems, 10th Revision, Australian Modification.

Supplementary Table 3. Unadjusted and adjusted relative risks of not achieving NMS on NAPLAN assessments and not completing high school for young people hospitalized with concussion compared to matched peers not hospitalized with any injury by school year, linked health and school performance data NSW, 2005–2018.

	RR	95%CI	ARR	95%CI
**NAPLAN assessment below NMS**				
Reading (n=3,685 matched pairs) [a]	1.36	1.13–1.63	1.40	1.17–1.67
Writing (n=2,121 matched pairs) [a]	1.37	1.10–1.70	1.42	1.17–1.73
Spelling (n=3,685 matched pairs) [a]	1.39	1.17–1.65	1.43	1.21–1.69
Grammar (n=3,685 matched pairs) [b]	1.35	1.14–1.59	1.39	1.18–1.64
Numeracy (n=3,685 matched pairs) [c]	1.26	1.02–1.56	1.30	1.05–1.62
**No high school completion**				
Year 10 (n=1,443 matched pairs) [d]	1.29	0.90–1.85	1.29	0.90–1.85
Year 11 (n=1,364 matched pairs) [d]	1.63	1.38–1.94	1.64	1.39–1.95
Year 12 (n=1,181 matched pairs) [d]	1.76	1.49–2.07	1.77	1.50–2.09

ARR: adjusted relative risk; NAPLAN: National Assessment Plan for Literacy and Numeracy; NSW: New South Wales; NMS: National Minimum Standard; RR: unadjusted relative risk.

[a] Generalized linear mixed model. Final fitted model included concussion status, sex, socioeconomic status, language background, parental education, school sector, and NAPLAN assessment year.

[b] Generalized linear mixed model. Final fitted model included concussion status, sex, language background, parental education, school sector, and NAPLAN assessment year.

[c] Generalized linear mixed model. Final fitted multilevel model included concussion status, sex, socioeconomic status, health conditions, parental education, school sector, and NAPLAN assessment year.

[d] Generalized linear model. Final fitted model included concussion status, sex, and socioeconomic status.

Supplementary Table 4. School performance outcomes for young people hospitalized with concussion and matched peers not hospitalized with any injury by school year, linked health and school performance data NSW, 2005–2018.

	Case		Comparison		*P* Value [a]
	**No.**	**%**	**No.**	**%**	
**NAPLAN reading assessment below NMS**					
Year 3 (n=1,049 matched pairs)	71	6.8	58	5.5	0.237
Year 5 (n=1,035 matched pairs)	94	9.1	61	5.9	0.006
Year 7 (n=932 matched pairs)	64	6.9	50	5.4	0.176
Year 9 (n=689 matched pairs)	59	8.6	43	6.2	0.100
**NAPLAN writing assessment below NMS**					
Year 3 (n=1,049 matched pairs)	65	6.2	38	3.6	0.006
Year 5 (n=1,035 matched pairs)	98	9.5	87	8.4	0.397
Year 7 (n=932 matched pairs)	124	13.3	107	11.5	0.232
Year 9 (n=689 matched pairs)	141	20.5	121	17.6	0.170
**NAPLAN spelling assessment below NMS**					
Year 3 (n=1,049 matched pairs)	79	7.5	53	5.1	0.019
Year 5 (n=1,035 matched pairs)	91	8.8	71	6.9	0.102
Year 7 (n=932 matched pairs)	87	9.3	72	7.7	0.214
Year 9 (n=689 matched pairs)	91	13.2	57	8.3	0.003
**NAPLAN grammar assessment below NMS**					
Year 3 (n=1,049 matched pairs)	81	7.7	54	5.2	0.016
Year 5 (n=1,035 matched pairs)	103	10.0	76	7.3	0.035
Year 7 (n=932 matched pairs)	95	10.2	77	8.3	0.150
Year 9 (n=689 matched pairs)	87	12.6	66	9.6	0.072
**NAPLAN numeracy assessment below NMS**					
Year 3 (n=1,049 matched pairs)	67	6.4	49	4.7	0.086
Year 5 (n=1,035 matched pairs)	70	6.8	58	5.6	0.274
Year 7 (n=932 matched pairs)	31	3.3	30	3.2	0.896
Year 9 (n=689 matched pairs)	25	3.6	16	2.3	0.154
**No high school completion**					
Year 10 (n=1,445 matched pairs)	67	4.6	52	3.6	0.160
Year 11 (n=1,366 matched pairs)	350	25.6	213	15.6	<.001
Year 12 (n=1,182 matched pairs)	388	32.8	221	18.7	<.001

NAPLAN: National Assessment Plan for Literacy and Numeracy; NSW: New South Wales; NMS: National Minimum Standard.

[a] Chi-squared test.
